# Electronic metal-support interactions in vacuum vs. electrolyte

**DOI:** 10.1038/s41467-020-15307-8

**Published:** 2020-03-19

**Authors:** Colleen Jackson, Graham Smith, Andrea E. Russell, Pieter Levecque, Denis Kramer

**Affiliations:** 1 Department of Chemistry, Imperial College London, Exhibition Road, London, SW7 2AZ UK; 2 HySA/Catalysis, Catalysis Institute, Department of Chemical Engineering, University of Cape Town, Corner of Madiba Circle and South Lane, Rondebosch, 7701 South Africa; 3 School of Chemistry, University of Southampton, University Road, Southampton, SO17 1BJ UK; 4 Engineering Sciences, University of Southampton, University Road, Southampton, SO17 1BJ UK; 5 Faculty of Mechanical Engineering, Helmut Schmidt University, 22043 Hamburg, Germany

**Keywords:** Electrocatalysis, Electrocatalysis, Fuel cells

**Replying to** Binninger *Nature Communications* 10.1038/s41467-020-15306-9

In our communication^1^, we have investigated Pt nanoparticles supported on high surface area carbons (Pt/C) and boron carbide composites (Pt/BC). We showed that purely electronic interactions between the nanoparticulate catalyst and its support have bearings on electrocatalytic activity for the oxygen reduction reaction in acidic media and catalyst stability. This has been achieved by interrogating the electronic states of the supported Pt catalysts in an electrochemical environment via X-ray absorption near edge structure (XANES), which showed a relatively more positive charge on Pt nanoparticles if supported on BC compared to very similar Pt nanoparticles supported on C. Similarity of the Pt nanoparticle size distribution and morphology was established by transmission electron microscopy (TEM) and extended X-ray absorption fine structure (EXAFS), respectively, to exclude other factors contributing to the electrocatalytic characteristics.

We also investigated the two catalysts ex situ under ultra-high vacuum (UHV) conditions by X-ray photoelectron spectroscopy (XPS), which has shown a shift to higher binding energies of the Pt 4f signal relative to the reference C 1s signal for the Pt/BC catalyst. Following the band filling argument put forward by Watanabe et al.^2^, we have tentatively interpreted this change with a relatively more negative charge of Pt supported on BC compared to Pt on C under UHV conditions. A fuller account of the reasoning is contained in the published reviewer file of our original communication.

This led us to point out that the relative state of charge of the Pt nanoparticles on the two supports (i.e., more negative or more positive) appears different under potentiostatic control in aqueous electrolyte than under UHV conditions. In an attempt to rationalise this observation, we have argued that the overall work function of the heterogeneous electrode surfaces should be different for the Pt/C and Pt/BC systems, which leads to a shift of the potential of zero charge (pzc)^3^ of the heterogeneous Pt/BC electrode surface relative to Pt/C. An additional positive charge has, therefore, to be accommodated by the Pt/BC electrode if held at the same potential, because it is further away from the pzc.

Binninger addressed this minor point^4^. In an attempt to show that “no inversion of the relative charge transfer between support and Pt nanoparticle can be deduced”, Binninger has put forward an electrostatic argument. He investigates the potential at which the nanoparticle electrolyte-facing, external surface has zero charge in the limit of infinitely strong screening (i.e., where the Debye length is much shorter than the catalyst particle size) in the electrolyte in detail and concludes that this potential is the same as is found for an extended Pt electrode under the same conditions. This result is unsurprising given that the assumption of infinite screening will quench all electrostatic interaction between nanoparticle and support through the electrolyte. Binninger then qualitatively expands his consideration to address weakly screening, purely dielectric interactions as well as chemical interactions between support and electrolyte and concedes that these have bearings on the charge held by the catalysts external interface and “potentially also change its sign”.

Below we will first attempt to clarify the definition of the potential of zero charge for heterogeneous electrodes used in our original communication, which is different from the quantity investigated by Binninger. We then proceed with analysing the charging behaviour of the nanoparticles and support and will show that this leads to conclusions that are incompatible with experimental observations of our systems, where the Debye length (0.1 M HClO_4_: *d*_OHP _≈ 0.6 nm; *λ*_dif_ > 2 nm)^5^ is of the same order of magnitude as the size of the nanoparticles (*r* ≈ 1.5 nm).

## Potentials of zero charge

First, we like to clarify that the potential of zero charge (pzc) that we have referred to in our original communication is distinct from the potential where the nanoparticles have zero charge that is the subject of Binninger’s consideration.

**Fig. 1:**
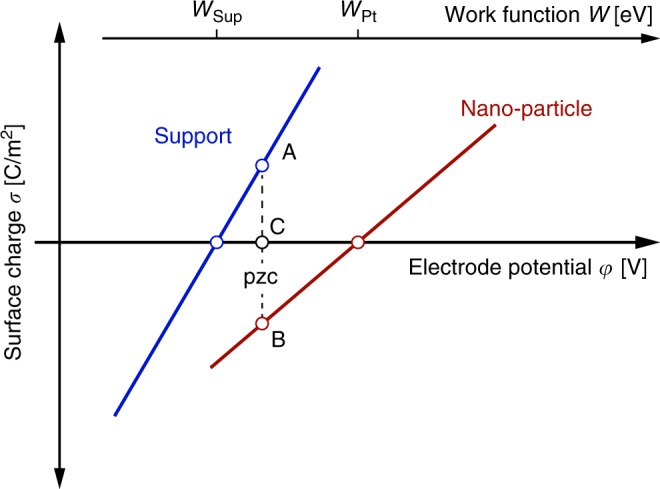
Idealised sketch of the potential of zero charge of a heterogeneous electrode. Shown are linearised charging curves per total surface area for support and nano-particle as a function of potential; the overall pzc is defined by equal but opposite charges on support (line AC) and nano-particle (line BC).

The diagram of Fig. 1 shows a simplified sketch of the charging behaviour of the nanoparticles and support. Following Binninger’s assumptions, we only consider here electrostatic interactions and assume that the charging is purely capacitive and linear in potential following the commonly used model from Bockris et al.^6^. This might not be justified in general as we will point out below, but it is useful to understand the implications of Binninger’s assumptions.

The nano-particle and support, in general, have different work functions. Hence, they will exchange charge until their Fermi levels are equilibrated when brought into contact with the forming dipoles resulting in a coverage-dependent change of overall work function^7^ and corresponding overall pzc. Under overall charge neutrality and assuming that charging across the direct catalyst-support interface is fully separable from charging across the electrolyte-facing interfaces, the magnitude of charge transferred from the support surface (line AC in Fig. 1) to the nanoparticle surface (line BC in Fig. 1) has to be equal at the pzc of the overall electrode. This clearly is a function of the support material and distinct from the potential where the catalyst nanoparticles have zero charge.

Figure 1 suggests that a lower work function of the support should shift the pzc towards more negative potentials, but the implied linear relationship could be an oversimplification. It is, therefore, valid to ask if the overall work function (and consequently the pzc) would also be framed by the work functions of the support and catalyst nanoparticle more generally. The overall work function of similar heterosystems has been shown theoretically^7^ and experimentally^8^ to fall in-between the work functions of the support and nanoparticle, giving us reason to believe that this relationship holds qualitatively more generally.

## Pt nanoparticle charging behaviour

As we have pointed out in our original communication and clarified above, this shift of pzc implies a more positive charge for the electrode with the lower work function support if the electrodes are held at the same potential. However, the question remains, how the additional charge is distributed across the catalyst and support? An important consequence of assuming full screening through the electrolyte is that the charge on the Pt nanoparticles as a function of voltage would be independent of the support material, not only at the potential where the nanoparticles carry zero charge as pointed out by Binninger, because the charge–voltage curve is the same for the nanoparticles in contact with the support and without. In contrast, our experimental results indicate that a larger fraction accumulates at the nanoparticles, but a general theoretical understanding of the charge distribution away from the pzc eludes us so far. Chemical and electrostatic interactions with the electrolyte and/or the electronic structure of catalyst and support could all be important factors.

We stress that the reasoning above reflects a highly idealised situation. As we have communicated recently elsewhere^9^, the charging behaviour of the Pt nanoparticles supported on BC as a function of voltage in acidic electrolyte is markedly different from the Pt nanoparticles supported on C. The electronic structure of Pt nanoparticles supported on BC and C was probed using XANES over a range of potentials. These experiments showed that at moderate potentials (below approximately 0.9 V vs. RHE), the Pt/BC catalyst has a higher d-band vacancy density than the Pt/C catalyst, while at higher potentials above 0.9 V vs. RHE the Pt/C catalyst showed more vacancies in the Pt d-band manifold, providing direct experimental evidence for a change of sign of the relative d-band filling between the two catalysts depending on potential.

Unfortunately, the situation is complicated by chemical interactions between catalyst and electrolyte at high potentials, and the relative trends in d-band vacancy should not be directly correlated with the state of charge of the nanoparticles at high potentials, as partial oxidation of the nanoparticles will contribute as well. The complex charging behaviour of Pt in aqueous electrolyte has recently been investigated in great detail by Eikerling and co-workers^10^ using a quantitative model that goes beyond that discussed by Binninger. They could show that the charging behaviour of metallic Pt cannot be assumed to be monotonic in aqueous electrolyte and that there is no unique potential of zero charge of metallic Pt due to the complexity of the interactions between Pt and aqueous electrolytes.

## Pt nanoparticle outer surface charge

Finally, we like to comment on Binninger’s assertion that “the majority of transferred charge is accumulated at the direct support-Pt interface and, therefore, unaffected by the external environment”. As we recently communicated elsewhere^9^, we used potentiostatic CO displacement measurements^11^ to estimate the surface charge of the Pt nanoparticles in electrochemical environments. Unfortunately, these measurements cannot be used to directly obtain the pzc, partially due to the common occurrence of charge-transfer chemisorption to form adsorbed hydrogen^12^. However, these measurements have shown displacement charges of about 60 μC/cm_Pt_^2^ for Pt/BC and 20 μC/cm_Pt_^2^ for Pt/C, respectively, in the double layer region (potentials of about 0.3 to 0.6 V vs. RHE) where chemisorbed hydrogen is unlikely to occur. Estimates for the pzc of extended Pt electrodes vary, but usually range from close to 0 V vs. SHE^12^ to values closer to 0.25 V vs. SHE^13^. Regardless, Pt electrodes in the electrolyte used (0.1 M HClO_4_) are typically reported to have a capacitance of around 15 μF/cm_Pt_^2^ ^11–13^, which would suggest surface charges no larger than 10 μC/cm_Pt_^2^ in the double layer region for unsupported Pt electrodes. Geometric effects^14^ might contribute to the larger capacitance of the supported catalysts relative to the expected capacitance of unsupported Pt, but are less relevant for the comparison of Pt/BC and Pt/C given their similar geometries. We, therefore, take the larger displacement charge of Pt/BC relative to Pt/C as an indirect indication of a shift of the pzc towards lower values for Pt/BC compared to Pt/C and further experimental evidence that electronic metal support interactions in electrochemical environments are not largely confined to the direct catalyst-support interface as conjectured by Binninger, but have bearings on the electrochemical properties of the catalyst-electrolyte interface. The dependence of CO stretching modes on local electric fields^15^ might provide convenient spectroscopic probes (e.g., through Infrared Reflection−Absorption Spectroscopy or Sum-Frequency Vibrational Spectroscopy^16^) to further study these surface charge effects.
